# The Impact of Sampling Season and Catching Site (Wild and Aquaculture) on Gut Microbiota Composition and Diversity of Nile Tilapia (*Oreochromis niloticus*)

**DOI:** 10.3390/biology10030180

**Published:** 2021-03-01

**Authors:** Negash Kabtimer Bereded, Getachew Beneberu Abebe, Solomon Workneh Fanta, Manuel Curto, Herwig Waidbacher, Harald Meimberg, Konrad J. Domig

**Affiliations:** 1 Institute of Food Science, University of Natural Resources and Life Sciences Vienna (BOKU), Muthgasse 18, 1190 Vienna, Austria; konrad.domig@boku.ac.at; 2 Department of Biology, Bahir Dar University, Bahir Dar, Post Code 79, Ethiopia; gech13@gmail.com; 3 Faculty of Chemical and Food Engineering, Bahir Dar Institute of Technology, Bahir Dar University, Bahir Dar, Post Code 26, Ethiopia; solworkneh@gmail.com; 4 Institute for Integrative Nature Conservation Research, University of Natural Resources and Life Sciences Vienna (BOKU), Gregor-Mendle-Straße 33, 1180 Vienna, Austria; manuel.curto@boku.ac.at (M.C.); meimberg@boku.ac.at (H.M.); 5 MARE−Marine and Environmental Sciences Centre, Faculdade de Ciências, Universidade de Lisboa, Campo Grande, 1049-001 Lisboa, Portugal; 6 Institute for Hydrobiology and Aquatic Ecosystems Management, University of Natural Resources and Life Sciences Vienna (BOKU), Gregor-Mendle-Straße 33/DG, 1180 Vienna, Austria; herwig.waidbacher@boku.ac.at

**Keywords:** aquaculture, 16S rDNA, fish, bacterial community, lake

## Abstract

**Simple Summary:**

The gut microbiota (all microbes in the intestine) of fishes is known to play an essential role in diverse aspects of their life. The gut microbiota of fish is affected by various environmental parameters, including temperature changes, salinity and diet. This study characterised the microbial composition in gut samples of Nile Tilapia collected from Lake Tana and the Bahir Dar aquaculture facility centre applying modern molecular techniques. The results show clear differences in the gut microbiota in fish from the Lake Tana and the ones from aquaculture. Further, also significant differences were observed on the composition of the gut microbiota across sampling months. Samples from the aquaculture centre displayed a higher diversity than the wild catch Nile tilapia from Lake Tana even though there is also an overlapping of the detected microbial groups. Overall, this is the first study on the effects of sampling season and catching site on the gut microbiota of Nile tilapia in Ethiopia. Future work will help to precisely explain the causes of these changes and their influence of the health and growth of Nile tilapia in Ethiopian lakes as well as under aquaculture conditions.

**Abstract:**

The gut microbiota of fishes is known to play an essential role in diverse aspects of host biology. The gut microbiota of fish is affected by various environmental parameters, including temperature changes, salinity and diet. Studies of effect of environment on gut microbiota enables to have a further understanding of what comprises a healthy microbiota under different environmental conditions. However, there is insufficient understanding regarding the effects of sampling season and catching site (wild and aquaculture) on the gut microbiota of Nile tilapia. This study characterised gut microbial composition and diversity from samples collected from Lake Tana and the Bahir Dar aquaculture facility centre using 16S rDNA Illumina MiSeq platform sequencing. Firmicutes and Fusobacteria were the most dominant phyla in the Lake Tana samples, while Proteobacteria was the most dominant in the aquaculture samples. The results of differential abundance testing clearly indicated significant differences for Firmicutes, Fusobacteria, Bacteroidetes and Cyanobacteria across sampling months. However, Proteobacteria, Chloroflexi, Fusobacteria and Cyanobacteria were significantly enriched in the comparison of samples from the Lake Tana and aquaculture centre. Significant differences were observed in microbial diversity across sampling months and between wild and captive Nile tilapia. The alpha diversity clearly showed that samples from the aquaculture centre (captive) had a higher diversity than the wild Nile tilapia samples from Lake Tana. The core gut microbiota of all samples of Nile tilapia used in our study comprised Firmicutes, Proteobacteria and Fusobacteria. This study clearly showed the impact of sampling season and catching site (wild and aquaculture) on the diversity and composition of bacterial communities associated with the gut of Nile tilapia. Overall, this is the first study on the effects of sampling season and catching site on the gut microbiota of Nile tilapia in Ethiopia. Future work is recommended to precisely explain the causes of these changes using large representative samples of Nile tilapia from different lakes and aquaculture farms.

## 1. Introduction

Nile tilapia (*Oreochromis niloticus*) is widely distributed in Africa and is one of the most preferred aquaculture fish species in the world [1]. It is a benthopelagic omnivorous fish that feeds on algae, aquatic plants, small invertebrates, detritus and associated bacterial films [2]. In fish farms, the major food items for Nile tilapia are commercial diets with a high protein content and aquafeeds formulated from plant and animal processing products and by-products, brewery wastes and poultry and fish by-products [3,4,5]. It is a fast-growing fish capable of tolerating a wide range of environmental conditions [6,7]. The optimum water temperature for rearing Nile tilapia is between 27 and 32 °C [8]. The fish begins to die when the water temperature drops to 11 °C, and it cannot survive below 8 °C [9].

The gut microbiota plays important roles in a wide range of biological processes of their host. They improve host health by facilitating nutrient and energy extraction through fermentation of nondigestible dietary components in the intestine [10]. Gut microbial communities also synthesise vitamins and amino acids [11]. Moreover, the gut microbiota inhibits the performance of pathogenic microbes and hence enhances the health of the fish [12]. The involvement of gut microbiota in fish nutrition, epithelial development, immunity and vulnerability to disease is well documented [11].

Season is considered as the major factor affecting the composition of gut microbiota in various fish species [13]. Al-Harbi and Naim Uddin [13] have reported seasonal variation in gut microbiota in the intestine of hybrid tilapia. In their study, the total viable counts (TVCs) of bacteria in the intestine varied between the early summer, summer, autumn and winter seasons. Moreover, seasonal variations in gut microbiota in farmed Atlantic salmon have been reported by Hovda et al. [14]. Similarly, the composition of intestinal lactic acid bacteria (LAB) has been observed to vary between seasons for Atlantic salmon [15], silver carp (*Hypophthalmichthys molitrix*), common carp (*Cyprinus carpio*), channel catfish (*Ictalurus punctatus*) and deep bodied crucian carp (*Carassius cuvieri*) [16]. Water temperature has been reported to affect LAB composition more than the physiological difference among the four fish species studied by Hagi et al. [16]. The composition of the gut microbiota in fish can also be influenced by other environmental parameters, such as salinity [17,18] and diet [19]. Moreover, captivity has also been reported to be one of the factors influencing the composition and diversity of the gut microbiota of fishes [20,21,22].

Lake Tana, the largest lake in Ethiopia, is situated on the basaltic plateau of the northwestern highlands of the country. Abundant wetlands, swamps, marshes and floodplains can be found all around the shores of the lake and its tributaries [23]. Lake Tana is well-known for its impressive diversity and unique fish species. Approximately 68% of the fish species in Lake Tana are endemic [24]. The large African barbs, Nile tilapia and African catfish are the most economically important fishes from the lake. Moreover, the lake is the source of the Blue Nile, a transboundary river of political importance.

Given the important roles of gut microbiota in host health, the assessment of gut microbiota may constitute an important aspect to manage the health of Nile tilapia and hence reduces production losses during aquaculture practices. Baseline information on the variations in gut microbiota in Nile tilapia sampled in different months representing different seasons is necessary to understand which microbial communities are dominant and most beneficial. However, there is no such study for Lake Tana, the lake with impressive fish diversity and uniqueness [24]. Therefore, in this study, we characterised the gut microbiota of Nile tilapia from Lake Tana and compared it with samples from a nearby aquaculture facility centre. We assessed the influence of sampling season on the Nile tilapia gut microbiota by analysing the samples collected in April, May, June, July and August representing dry season, pre-rainy season and main-rainy season. Sedimentation, increased trend of eutrophication and toxigenic cyanobacteria are reported as major problems of the Lake Tana basin [25] and these factors believed to show seasonal fluctuation due to the variation of amount of rainfall in each season. In addition, differences between the gut microbiota composition of wild fish from Lake Tana and farm fish from aquaculture facility centre were investigated. We hypothesised that the gut microbiota will change throughout the year as well as differ depending on the habitat, in this case natural versus artificial water bodies. This would support findings that differences on environmental variables play a role in shaping the intestinal microbiota of fish [12]. This information could be used to enhance the economic benefits of aquaculture since it enables proper feed composition and enrichment with the necessary probiotics.

## 2. Methods and Materials

### 2.1. Specimen Collection and Sampling Sites

**Figure 1:**
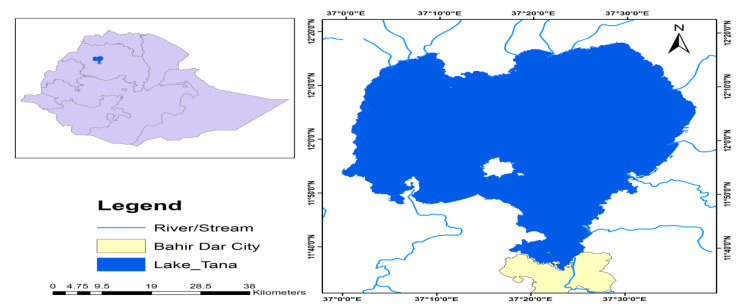
A map showing the sampling site of Nile tilapia in Ethiopia.

The specimens were collected from Lake Tana and the Bahir Dar aquaculture facility centre at the Amhara Regional Agricultural Research Institute (ARARI), which is located close to Lake Tana. The aquaculture centre gets water from the lake. Lake Tana is a high-altitude lake (1800 m above sea level) and covers a surface area of 3200 km^2^. Its trophic status changed to mesotrophic and eutrophic due to nutrient loads [26]. Lake Tana is a shallow lake with an average depth of 8 m and a maximum depth of 14 m. Lake Tana basin lies between latitudes 10°95′ and 12°78′ N and longitudes 36°89′ and 38°25′ E. The climate of Lake Tana is divided roughly into four seasons: the main rainy season (July–September), dry season (December–April), pre-rainy season (May–June) and post-rainy season (October–November) [27]. The mean annual rainfall of the catchment area is approximately 1280 mm [28]. The lake is fed by several tributary rivers, of which four are permanent: Megech, Rib, Gumara and Gilgel Abay and the Blue Nile is the only out flowing river (Figure 1). 

### 2.2. Fish Sampling and Processing

A total of 47 adult male Nile tilapia samples were collected from the landing site of Lake Tana, and seven samples were supplied by the Bahir Dar aquaculture facility centre (Table 1). For the Lake Tana samples, specimen collection was performed on a monthly basis from April to August 2018. The aquaculture samples were collected in August 2018. For comparison of the wild population with the aquaculture samples, only samples collected in August from Lake Tana were used. All fish samples after collection were treated similarly as previously reported [29]. Briefly, the fish were killed by high doses of clove oil [30] and aseptically dissected after washing the outer surfaces and instruments using 70% ethanol. The hindgut luminal contents were collected as described by Ghanbari et al. [31] and placed in sterile screw cap tubes containing sterile phosphate-buffered saline and glycerol. The samples were stored at −20 °C until further processing.

### 2.3. DNA Extraction, PCR Amplification of 16S rRNA and Amplicon Sequencing

DNA extraction of gut contents was performed using the PowerFecal^®^ DNA Isolation Kit (Qiagen, Hilden, Germany) with some modifications. Two-step PCR was conducted to amplify the V3–V4 hypervariable region of the 16S rRNA gene for the Illumina MiSeq system (Illumina, San Diego, CA, USA) following Shokralla et al. [32]. Details regarding DNA extraction, the two PCR steps and PCR product purification steps have been published previously [29]. High-throughput sequencing analysis of bacterial rRNA genes was performed using an Illumina MiSeq paired-end (PE) 300 sequencing platform (San Diego, CA, USA) at the Genomics Service Unit, Ludwig-Maximilian’s-Universität München, Germany. The run was performed as a joint run together with other libraries.

### 2.4. Sequence Data Processing

Sequences were quality controlled with Cutadapt v. 0.11.1 [33] by removing regions matching the adapter sequences and the remaining downstream sequence with the default settings. Regions with low sequence quality were excluded with the same program with the sliding window approach, allowing a minimum quality of 30. Trimmed reads with a length below 200 bp were excluded. Paired reads were merged with PEAR v. 0.9.4 [34] with the default settings, and overlapping sequences smaller than 200 bp were deleted. Merged reads were checked if they contained the correct primer sequence information with an in-house script presented in Curto et al. [35] with small modifications. A maximum of two mismatches between primer sequences and reads were allowed, and matching regions were trimmed out. USEARCH 6.0 was used to detect chimaeras based on the RDP pipeline [36]. The reads were then clustered into operational taxonomic units (OTUs) based on 97% identity using USEARCH. The OTU table was created by mapping reads to OTUs with the ‘otutab’ command in USEARCH. Taxonomy was assigned for the generated OTUs using the Ribosomal Database Project (RDP) classifier (Naive Bayesian rRNA classifier) [37]. Data filtering was performed to remove low-quality or uninformative features using default minimum counts of 4 and 20% prevalence in samples on MicrobiomeAnalyst [38] to improve the downstream statistical analysis. Data rarefaction to a minimum library size was performed to address the variability in sampling depth before further downstream processing.

### 2.5. Data Analysis

The gut microbiota structure between sampling months and habitat was assessed using the nonparametric univariate Mann–Whitney test. Comparison of bacterial taxa abundance between sampling months and between Lake Tana (collected in August) and the Bahir Dar aquaculture facility centre was performed using the linear discriminant analysis effect size (LEfSe). A nonparametric Kruskal–Wallis (KW) sum-rank test was used to detect features with significant differential abundance with respect to the groups compared, followed by linear discriminant analysis (LDA) to estimate the effect size of each differentially abundant feature [39]. Core microbiome analysis was performed using the core function in the R package microbiome as described in MicrobiomeAnalyst [38]. The ‘core microbiota’ refers to a set of abundant microbial communities present in all individuals from the same species [40]. To detect the core microbiome, 20% prevalence and 0.01% relative abundance were used.

Alpha diversity and beta diversity statistics were performed using the phyloseq package as used in MicrobiomeAnalyst [38]. The alpha diversity of each sample was assessed using the observed species, Shannon index and Simpson index. The observed species calculates the actual number of unique taxa observed, while Shannon and Simpson consider both evenness (abundance of organisms) and richness (number). Beta diversity represents the variation of microbial communities between samples. The dissimilarity matrix was calculated using compositional-based Bray Curtis distance method. To visualise the dissimilarity matrix in lower dimensions, principal coordinate analysis (PCoA) was used. The statistical significance of the clustering pattern in ordination plots was evaluated using permutational multivariate analysis of variance (PERMANOVA), analysis of group similarities (ANOSIM) and homogeneity of group dispersions (PERMDISP).

## 3. Results

A total of 130,848 sequences were obtained from the tilapia gut microbiome by Illumina MiSeq sequencing. Overall, 1055 OTUs were identified from all samples analysed. Rarefaction curves approached the saturation phase in all samples (Supplementary Figure S1). 

### 3.1. Temporal Comparison of Gut Microbiota of Wild Fish from the Lake

**Figure 2:**
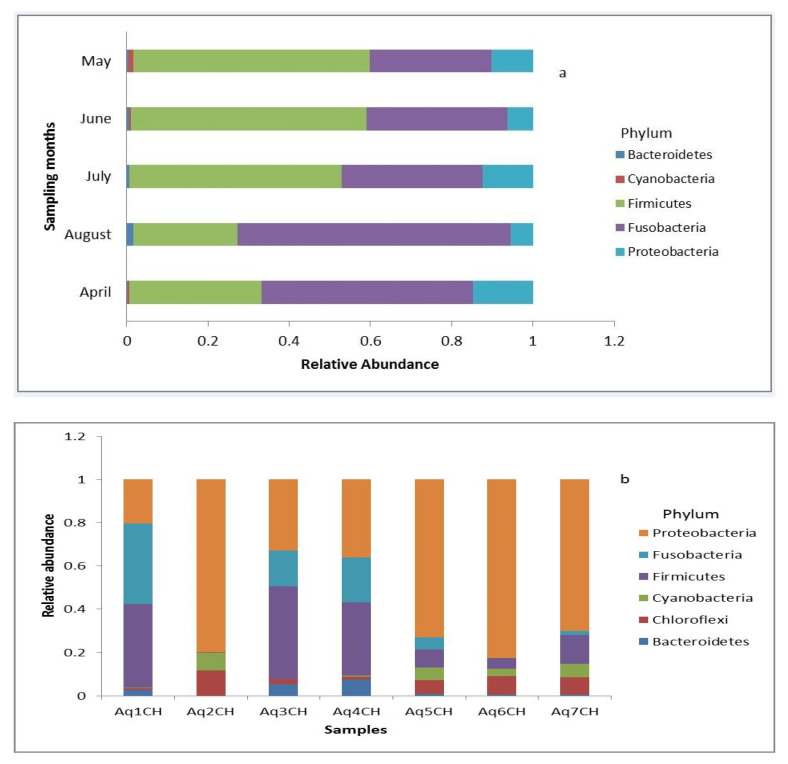
Taxonomic composition of the bacterial community at the phylum level using a stacked plot. (**a**) Lake Tana samples shown on a monthly basis. (**b**) Bahir Dar aquaculture facility centre samples.

The gut bacteria of 47 Nile tilapia representing five months of sampling were examined to characterise their structure and to reveal the temporal differences between them. In total, five phyla representing 19 genera were obtained from the analysis (Figure 2a, Supplementary Figure S2). At the phylum level during May, June and July, the gut microbiota was dominated by Firmicutes followed by Fusobacteria. However, in April and August, Fusobacteria was dominant over Firmicutes. In each month, Bacteroidetes and Cyanobacteria were less abundant (Figure 2a). At the genus level, the gut microbiota was dominated by *Cetobacterium*, *Clostridium_sensu_stricto* and *Clostridium_XI* (Supplementary Figure S2).

**Figure 3:**
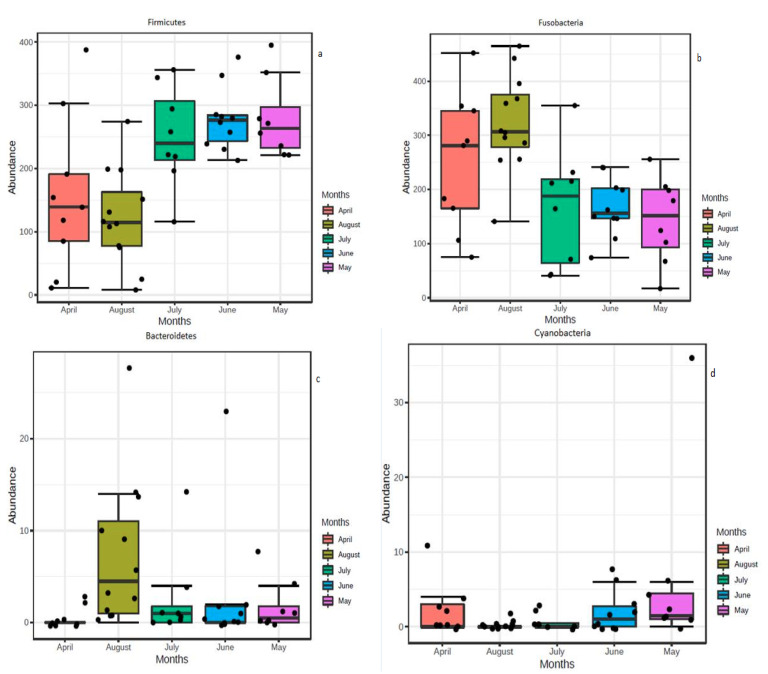
Comparative abundances of all the Lake Tana samples identified by univariate nonparametric analysis (Mann–Whitney test) based on a single grouping variable at phylum level. The black points represent individual samples. Features are considered to be significant based on their adjusted p-value = 0.05. All phyla showed significant *p*-value. (**a**) Phylum Firmicutes, (**b**) Phylum Fusobacteria, (**c**) Phylum Bacteroidetes and (**d**) Phylum Cyanobacteria.

There was a significant difference in terms of abundance for some bacterial groups among sampling periods. The results of differential abundance testing (Mann-Whitney test) clearly indicated significant differences for the phyla Firmicutes (*p*-value: 0.000444), Fusobacteria (*p*-value: 0.001407), Bacteroidetes (*p*-value: 0.013789) and Cyanobacteria (*p*-value: 0.034771) across sampling months. The abundance of Fusobacteria was higher during April and August, and Firmicutes was lower during these months (Figure 3a,b). Bacteroidetes were higher in August (Figure 3c), and a relatively higher Cyanobacteria abundance was observed in May (Figure 3d). At the genus level, a total of 12 significantly different taxa were detected from Lake Tana across the sampling months (Supplementary Table S1).

**Figure 4:**
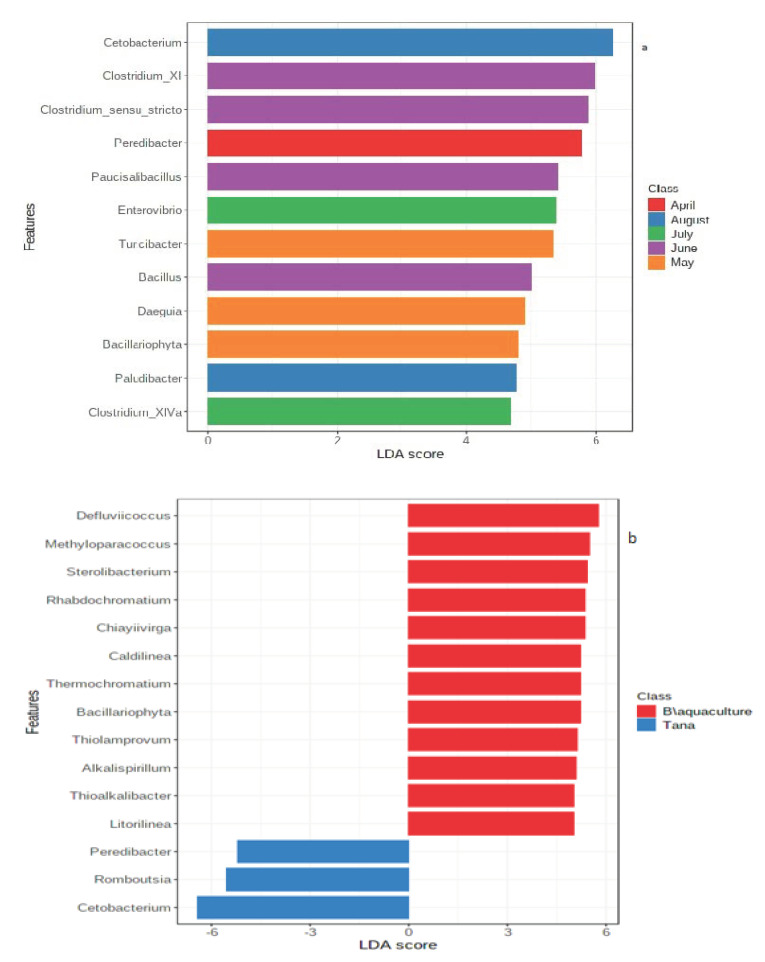
Graphical summary of important taxa identified by linear discriminant effect size (LEfSe) analysis at the genus level. (**a**) Lake Tana samples based on sampling months and (**b**) comparison of Lake Tana and Bahir Dar aquaculture facility centre samples. Horizontal bars represent the effect size for each taxon. Features are considered to be significant based on their adjusted *p*-value = 0.05.

Linear discriminant (LDA) effect size (LEfSe) analysis of the gut bacteria of Nile tilapia from Lake Tana at a default logarithmic LDA score of 2 showed that the taxon contributing most to the dissimilarity (effect size) for April was *Peredibacter.* Likewise, for May, *Turicibacter*, *Daeguia* and *Bacillariophyta*, for June, *Clostridium_XI*, *Clostridium_sensu_stricto*, *Paucisalibacillus* and *Bacillus*, for July, *Enterovibrio* and *Clostridium_XlVa* and for August, *Cetobacterium* and *Paludibacter* were found as taxa contributing most to the dissimilarity (Figure 4a).

**Figure 5:**
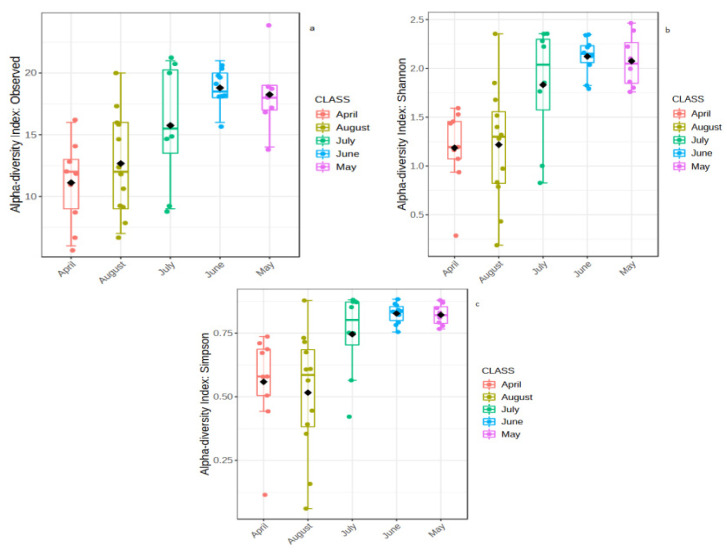
Alpha diversity measures at the OTU level are represented as boxplots for Lake Tana samples (**a**–**c**). Each boxplot represents the diversity distribution of a group present within the Months class. (**a**) Observed, (**b**) Shannon index and (**c**) Simpson index.

Analysis of the alpha diversity (observed, Shannon and Simpson indices) of the gut microbiota of samples from Lake Tana showed that the gut microbiota diversity varied significantly across the sampling months (*p*-values: 0.00020397, 0.00017971 and 8.96×10^-05^, respectively) (Figure 5a–c). We found that April and August had lower diversities than the other sampling months.

**Figure 6:**
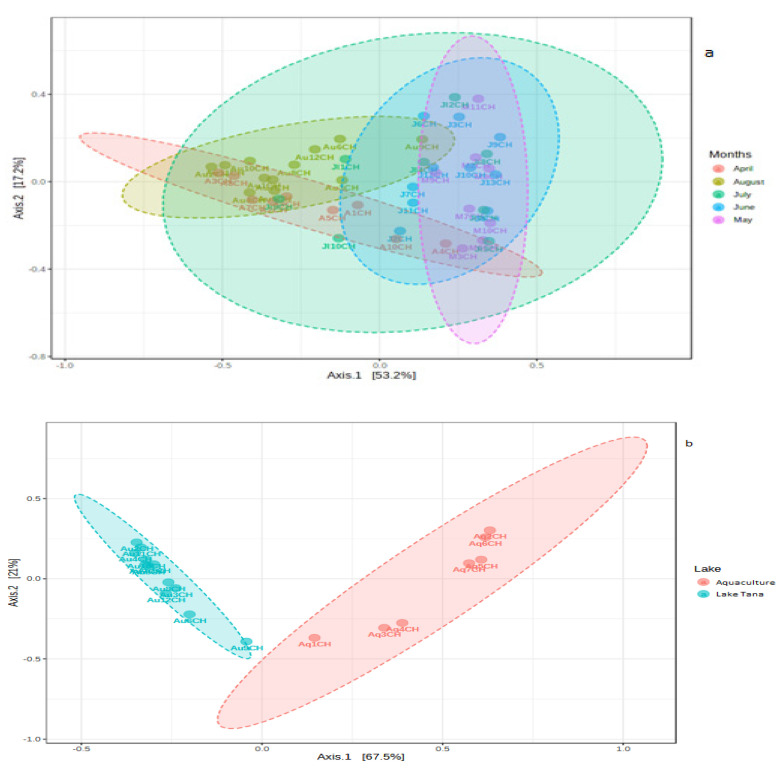
PCoA plot using Bray distance. The explained variances are shown in brackets. (**a**) PCoA plot for the Lake Tana samples based on the sampling months. (**b**) PCoA plot for the comparison of the Lake Tana and Bahir Dar aquaculture facility centre samples.

To visually display patterns of beta diversity, principal coordinate analysis (PCoA) plots were made using the Bray-Curtis index distance method. Beta diversity analysis revealed a clear separation of samples according to sampling months (from April to August) with a global *p*-value < 0.001 for PERMANOVA (Figure 6a). Furthermore, statistical analysis of beta diversity across the samples showed significant divergence of the microbial communities present in the gut across fish sampling months because the ANOSIM tests indicated a significant difference (*R*: 0.46066; *p*-value < 0.001). In addition, the nonsignificant results of the PERMDISP test of the sampling months (PERMDISP *F*-value: 2.672; *p*-value: 0.045007) indicated that group dispersions were similar to each other.

**Figure 7:**
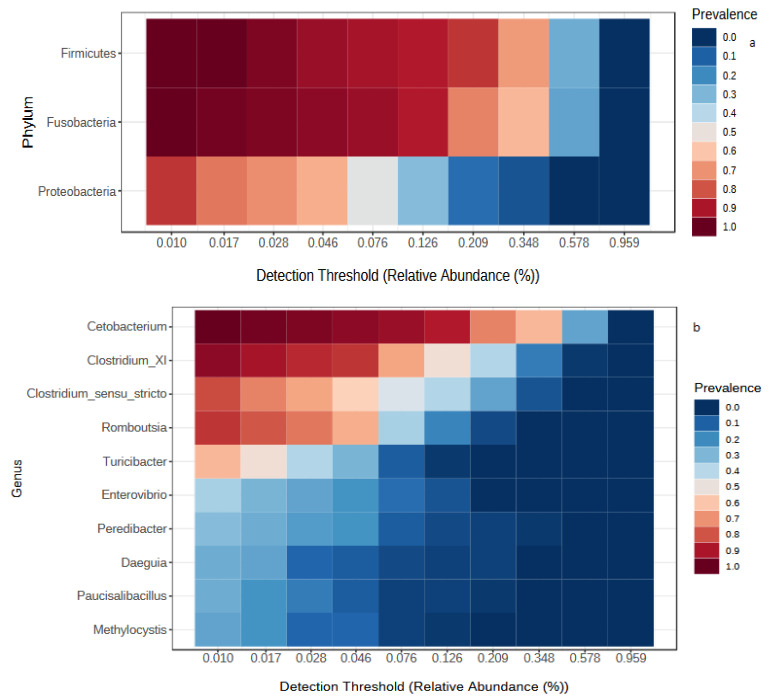

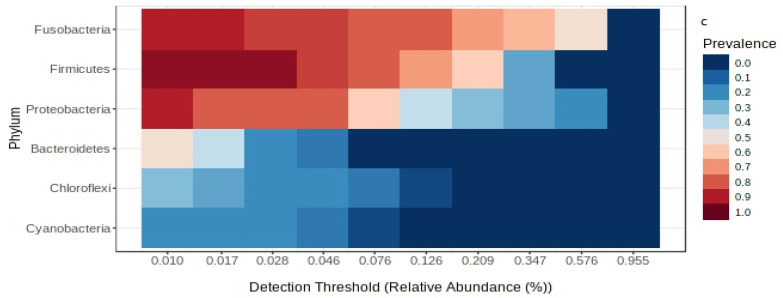
Heatmap showing the core microbiota. (**a**) Core phyla of all samples used in this study, (**b**) core genera of all samples, (**c**) core phyla from the comparison of the Lake Tana and aquaculture facility centre samples.

In our study, the core microbiota of all samples from Lake Tana and the Bahir Dar aquaculture facility centre comprised three phyla (Firmicutes, Proteobacteria and Fusobacteria) (Figure 7a). The number of core genera presented in all samples was 10 (Figure 7b), and the number of core genera for each month varied from 7 to 14 (Supplementary Figure S5).

### 3.2. Comparison of Gut Microbiota between Wild and Captive Fish

To assess the gut microbiota differences between wild and aquaculture Nile tilapia samples, samples of Lake Tana were compared with Bahir Dar aquaculture facility centre samples. As indicated in Figure 2b and Figure S3, the aquaculture samples were dominated by Proteobacteria, whereas the lake was dominated by Fusobacteria.

Between the samples from the Lake Tana and the Bahir Dar aquaculture facility centre, four phyla, Proteobacteria (*p*-value: 3.97×10^-05^), Chloroflexi (*p*-value: 0.000203), Fusobacteria (*p*-value: 0.000616) and Cyanobacteria (*p*-value: 0.0009), were found to be significantly different in Mann–Whitney test. The abundances of Proteobacteria, Chloroflexi and Cyanobacteria were higher in samples taken from the aquaculture centre than those taken from Lake Tana, and in contrast, the abundance of Fusobacteria was found to be higher in the Lake Tana samples (Supplementary Figure S4). A total of 30 significantly different genera were found from the comparison of samples from Lake Tana and the aquaculture facility centre (Supplementary Table S2).

The taxa found by LEfSe to be differentially abundant between samples of Lake Tana and aquaculture facility centre are shown in Figure 4b. The genera *Cetobacterium*, *Romboustia* and *Peredibacter* were significantly enriched in Lake Tana. On the other hand, genera such as *Defluviicoccus*, *Methyloparacoccus* and *Sterolibacterium* were significantly more abundant in the aquaculture samples. 

To assess the effect of the environment on gut microbiota diversity, samples from Lake Tana were compared with samples from the Bahir Dar aquaculture facility centre. The alpha diversity (observed *p*-value: 0.00043447, Shannon *p*-value: 3.96×10^-05^ and Simpson index *p*-value: 3.96×10^-05^) clearly showed that samples from the aquaculture facility centre had higher diversity than the wild Nile tilapia samples from Lake Tana (Supplementary Figure S6a–c).

The Beta diversity analysis revealed a clear separation of samples from Lake Tana and the Bahir Dar aquaculture facility centre (PERMANOVA *F*-value: 26.286; *R*-squared: 0.60726; *p*-value < 0.001) (Figure 6b). The ANOSIM test also indicated a significant difference between the two groups of samples (*R*: 0.93432; *p*-value < 0.001). In addition, the PERMDISP test showed a nonsignificant result (*F*-value: 2.3542; *p*-value: 0.14334). From this, the PERMANOVA result was found to be due to the average community composition differences.

From the comparison of Lake Tana and aquaculture facility centre samples, a total of 26 core genera were detected (Supplementary Figure S7). At the phylum level, the phyla Fusobacteria, Firmicutes, Proteobacteria, Bacteroidetes, Chloroflexi and Cyanobacteria were detected (Figure 7c).

## 4. Discussion

### 4.1. Overall Core Microbiota Composition

The gut microbiota of fish is involved in the digestion of food materials and can influence the nutrition, growth, reproduction, general population dynamics and health status of the host fish [41]. These microbial communities are sensitive to rearing environments [42]. In this study, gut microbiota of Nile tilapia from Lake Tana and aquaculture centre were investigated, considering potential variation with respect to season and catching site. 

The concept of a core gut microbiota has been suggested for certain fish species [40,43,44]. Trophic level, habitat and host phylogeny are reported as the major determinant factors for the core gut microbiota of fishes [17]. The core gut microbiota of all samples of Nile tilapia used in our study comprised Firmicutes, Proteobacteria and Fusobacteria. Similar to our study, Fusobacteria, Firmicutes and Proteobacteria represented the dominant components in the gut microbiota of Eastern African Cichlid Fishes [43]. The core gut microbiota of Nile tilapia from Lake Awassa and Chamo in Ethiopia was dominated by Proteobacteria, Firmicutes, Fusobacteria, Cyanobacteria and Actinobacteria [29]. All these core phyla have been previously reported as part of gut microbiota and hence have roles in the biology of fishes. *Proteobacteria* are often facultatively or obligately anaerobic and capable of tolerating a range of toxic conditions and are thought to contribute to the homeostasis of the anaerobic environment of the gut [45]. Firmicutes are involved in the fermentation of dietary fibres and regulate intestinal dietary fat absorption [46,47]. Fusobacteria are butyrate-producing anaerobic bacteria that are capable of fermenting amino acids and carbohydrates [48]. Due to the production of butyric acid in the gut, Fusobacteria possess immunomodulatory and anti-inflammatory properties [49]. In our study, the dominant genus from Fusobacteria was found to be *Cetobacterium*, which can produce vitamin B12 [50]. Enrichment of these taxa in the gut might solve deficiency of this vitamin in the diet. This might be the reason why juvenile tilapia had no dietary vitamin B_12_ requirement [51].

### 4.2. Seasonal Variation of Gut Microbial Communities

The results of this study showed that the gut microbiota of Nile tilapia from Lake Tana were dominated by the phyla Firmicutes and Fusobacteria. These results were in agreement with the findings of a gut microbiota study performed on Nile tilapia of Lake Nasser in Egypt, where they reported that Fusobacteria was the dominant phylum [52]. Ray et al. [53]; however, in their study, Firmicutes were less abundant, contradicting our results where they were the dominant phylum along with Fusobacteria. Inconsistency among studies on the dominant phyla showed, varying between Firmicutes [29,54] and Proteobacteria, Firmicutes and Cyanobacteria [55]. At the genus level, the gut microbiota was dominated by *Cetobacterium*, *Clostridium_sensu_stricto* and *Clostridium_XI*, which was different from other studies. Lukassen et al. [56] showed *Cetobacterium* as one of the predominant genera from the intestinal digesta of tilapia from a Brazilian reservoir. The observed discrepancies in microbiota composition may be due to several factors, including rearing environmental conditions, diet composition and genetic lineage. The gut bacterial communities of Nile tilapia larvae were significantly affected by the rearing environment (recirculating or active suspension systems) [42]. Diet type influences the diversity and difference of gut bacterial community of tilapia [57,58]. Moreover, a strong correlation between genotype and gut microbial assemblages in fish were reported recently [59].

Our data further confirm the temporal variation in terms of bacterial abundance in accordance with a number of previous studies on catfish [60] and Nile tilapia [61]. Our study indicated significant differences in the phyla Firmicutes, Fusobacteria, Bacteroidetes and Cyanobacteria across sampling months. At the genus level, a total of 16 significantly different taxa were detected (Supplementary Table S1). The abundance of Firmicutes was lower in April and August, whereas, for Fusobacteria, the reverse occurred (Figure 3a,b). The possible explanations might be the differences in nutrient inputs to the lake and possibly the variations in the availability of food sources in the water bodies during different months of the year. Seasonal fluctuation of nutrient loading in Lake Tana was reported recently [62]. Significant temporal differences in zooplankton abundance in Lake Tana were reported by Dejen et al., [63] and the highest densities of zooplankton were recorded during the dry season (November–April). Moreover, a significant difference in phytoplankton biomass during the sampling months was reported from Lake Tana [64]. Similarly, Wondmagegne et al. [65] found that the phytoplankton density in Lake Tana reached the highest points during the dry season and the pre-rainy season. Since Nile tilapia is an omnivorous fish capable of feeding both phytoplankton and zooplankton, the abundance of these food sources may be the reason for the seasonal variation in its gut microbiota.

In our study, the genera *Cetobacterium* (Fusobacteria) and *Paludibacter* (Bacteroidetes) were found to be the main contributors to the variation in August (Figure 4a). *Cetobacterium* is a microaerotolerant bacterium that is able to digest carbohydrates and peptides [53]. *Paludibacter* has been reported to have a fermentative metabolism and is able to utilise various sugars [66]. In addition, from Firmicutes, various taxa (both aerobic and anaerobic) have been found to be the main contributors to the variation and from this, it may be noted that there is a synergetic interaction in the gut. Firmicutes are involved in the degradation of dietary fibres [46]. The high abundance of Cyanobacteria observed likely supports their importance as food sources. Cyanobacteria are known to be important food sources for Nile tilapia [67,68]. The taxon contributing the most to this phylum was *Bacillariophyta*, which corresponds to chloroplasts of diatoms. A study by Mohamed et al. [67] indicated that *Bacillariophyta* was one of the main taxa present from cyanobacteria in the faecal samples of Nile tilapia, indicating consumption of the others as food. Diatoms constitute part of the biofilm present in freshwater systems [69], which given the detritivores feeding behaviour of Nile tilapia may be an import source of nutrients. As shown by our results they are affected both by season and aquaculture activity. Thus it is important to evaluate their importance for Nile Tilapia fitness.

The monthly variation in gut microbiota diversity observed could be associated with the availability of food sources and physicochemical variation in the lake. The effect of the rearing environment on the gut microbiota of Nile tilapia was reported by Giatitis et al. [42], and likewise, temporal variations in plankton abundances in Lake Tana have been reported [65]. A culture-dependent study on farm *O. niloticus* showed that bacterial flora are more diverse in the autumn and spring seasons than in the winter season [61]. Diversity and seasonal changes in gut lactic acid bacteria were also recorded by Hagi et al. [16] for catfish and carp. The effect of seasonality on the diversity of the gut microbiota of Tench (*Tinca tinca* L.) has also been reported by Dulski et al. [70].

### 4.3. Comparison of Wild and Captive Fish Gut Microbiota

In our study, the aquaculture samples were dominated by Proteobacteria. Similar to our study, Proteobacteria was reported as the dominant phylum in the gut of genetically improved farmed tilapia [55] and juvenile *O. niloticus* [71]. Proteobacteria is a major phylum of Gram-negative bacteria that plays a key role in preparing the gut for colonization by strict anaerobes by consuming oxygen and lowering the redox potential in the gut [72]. The higher abundance of Proteobacteria in the farm could be related to the high abundance of Proteobacteria in the aquaculture environment, which has been reported to be involved in the degradation of organic matter and nitrogen fixation [73,74,75].

In this study, there were significant differences between samples from Lake Tana and the Bahir Dar aquaculture facility centre in terms of the abundance of the phyla Proteobacteria, Chloroflexi, Fusobacteria and Cyanobacteria. In addition, a total of 30 significantly different genera were detected (Supplementary Table S2). The abundances of Proteobacteria, Chloroflexi and Cyanobacteria were higher in samples from the aquaculture facility centre than in those from Lake Tana (Supplementary Figure S4). Moreover, the study indicated unique taxa for both groups of samples (Figure 4b). Similar findings were reported by Dehler et al. [20] for a comparison of Atlantic Salmon parr between samples from a recirculating aquarium and open loch. In their study, the phyla Chloroflexi, Chloroplast, Tenericutes and Verrucomicrobia were only found in samples from the recirculating aquarium. According to Delport et al. [76], in the gut of Australian sea lion, Proteobacteria contributed more to captive than to wild Australian sea lion populations.

The alpha diversity clearly showed that the samples from the aquaculture facility centre had a higher diversity than the wild Nile tilapia samples from Lake Tana (Supplementary Figure S6a–c). This could be due to the different types of feed and habitats of the aquaculture and lake. The effect of different dietary nutrient compositions and form (live feeds or pelleted diets) on gut microbiota of fish was reviewed by Ringo et al. [77]. In contrast to our findings, the gut of wild Atlantic Salmon parr [20], Killifish *Fundulus heteroclitus* [21], flatfish *Paralichthys adspersus* [22] and Malaysian Mahseer *Tor tambroides* [78] had a higher microbial diversity than the farm specimens. This has been reported to vary depending on the animal [79]. For example, in mammals, the alpha diversity of gut bacteria increases, remains consistent or declines in captivity [79]. The authors mentioned that host traits, such as gut physiologies, dietary alteration, reduced contact with a social network and mode of feeding, are likely to influence the gut bacteria stability or change it in captivity in studied mammals. From the beta-diversity analysis, we observed that captivity determined the bacterial community clustering in the gut microbiota of Nile tilapia. In accordance with this, substantial differences in intestinal microbial community composition and diversity were observed between wild and farm Salmon [80]. 

## 5. Conclusions

To the best of our knowledge, this is the first characterization of the gut microbiota of Nile tilapia using a 16S rRNA sequencing approach for Lake Tana. This study indicates evidence of an impact of season and captive effect on the gut microbiota of Nile tilapia. In our study, the diversity and composition of bacteria associated with the gut of Nile tilapia varied between sampling months and habitat types (wild and aquaculture centre). Firmicutes, Proteobacteria and Fusobacteria were abundant in the guts of all Nile tilapia samples, indicating that these phyla are members of the core microbiota. To precisely explain the role of seasonality, rearing habitat, physico-chemical parameters of the environment and food source diversity in shaping gut microbiota composition and diversity, further studies are needed with large representative samples of Nile tilapia from different lakes and aquaculture farms. However, this study significantly extends the base knowledge of the effect of seasonality and captivity on the gut microbiota of Nile tilapia.

## Figures and Tables

**Table 1:** Samples used in this study.

Sampling Site	Sampling Months	No. of Samples	Season
Lake Tana	April	9	Dry season
Lake Tana	May	8	Pre-rainy season
Lake Tana	June	10	Pre-rainy season
Lake Tana	July	8	Main rainy season
Lake Tana	August	12	Main rainy season
Bahir Dar aquaculture centre	August	7	Main rainy season

## Data Availability

The raw sequences have been deposited to NCBI Sequence Read Archive(SRA), under the BioProject ID PRJNA705209.

## Supplementary Materials

The following are available online at https://www.mdpi.com/2079-7737/10/3/180/s1. Figure S1: Rarefaction curve. Figure S2: Taxonomic composition of bacterial community of Lake Tana samples at Genus level using stacked plot. Figure S3: Taxonomic composition of the bacterial community at the phylum level using a stacked plot of aquaculture samples and Lake Tana samples collected in August. Figure S4: Core microbiota analysis of Lake Tana samples at genus level for each sampling months.(a) April, (b) May, (c) June, (d) July, (e) August. Figure S5: Core microbiota analysis of Lake Tana and aquaculture facility centre at genus level. Figure S6: Alpha diversity measured at the OTU level across all the samples for comparison of Lake Tana and the Bahir Dar aquaculture facility centre. Each sample is coloured based on the sources of the samples. (a) Observed, (b) Shannon index, and (c) Simpson index. Figure S7: Core microbiota analysis of Lake Tana and aquaculture facility center at Genus level.Table S1: Important features identified by Univariate nonparametric analysis (Mann-Whitney test) at Genus level from Lake Tana samples. Table S2: Important features identified by Univariate nonparametric analysis (Mann-Whitney test) at Genus level from comparison of Lake Tana and Bahir Dar aquaculture facility center.
