# Supplementary material for: The Impact of Sampling Season and Catching Site (Wild and Aquaculture) on Gut Microbiota Composition and Diversity of Nile Tilapia (Oreochromis niloticus)
Source: Biology (Basel). 2021 Mar 1;10(3):180. doi: 10.3390/biology10030180 (PMC8001861; doi:10.3390/biology10030180)
Supplement: Supplementary file 1 [file biology-10-00180-s001.pdf]

biology

MDPI

Supplementary Figures and Tables

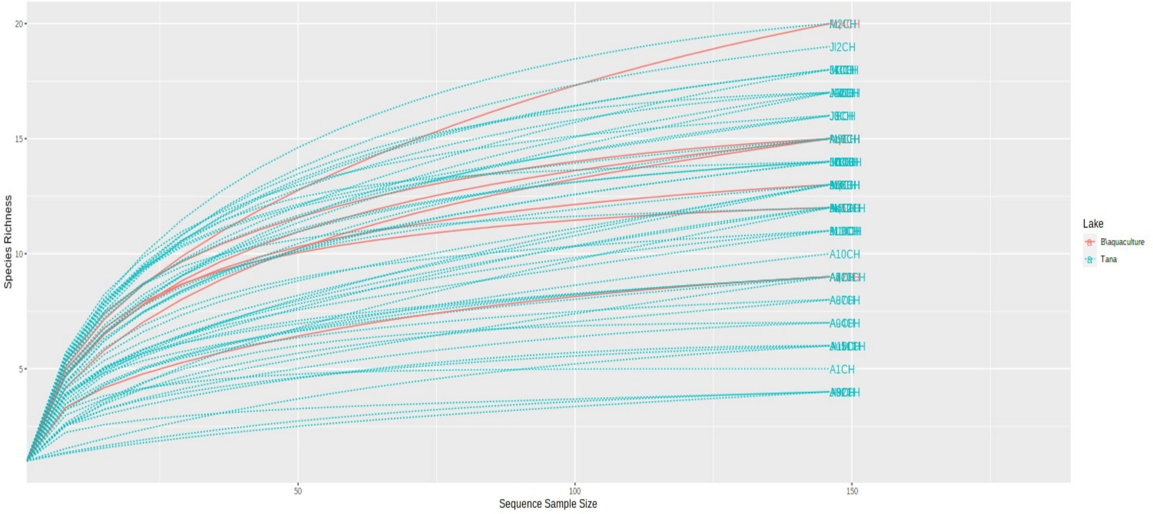

Figure S1. Rarefaction curve.

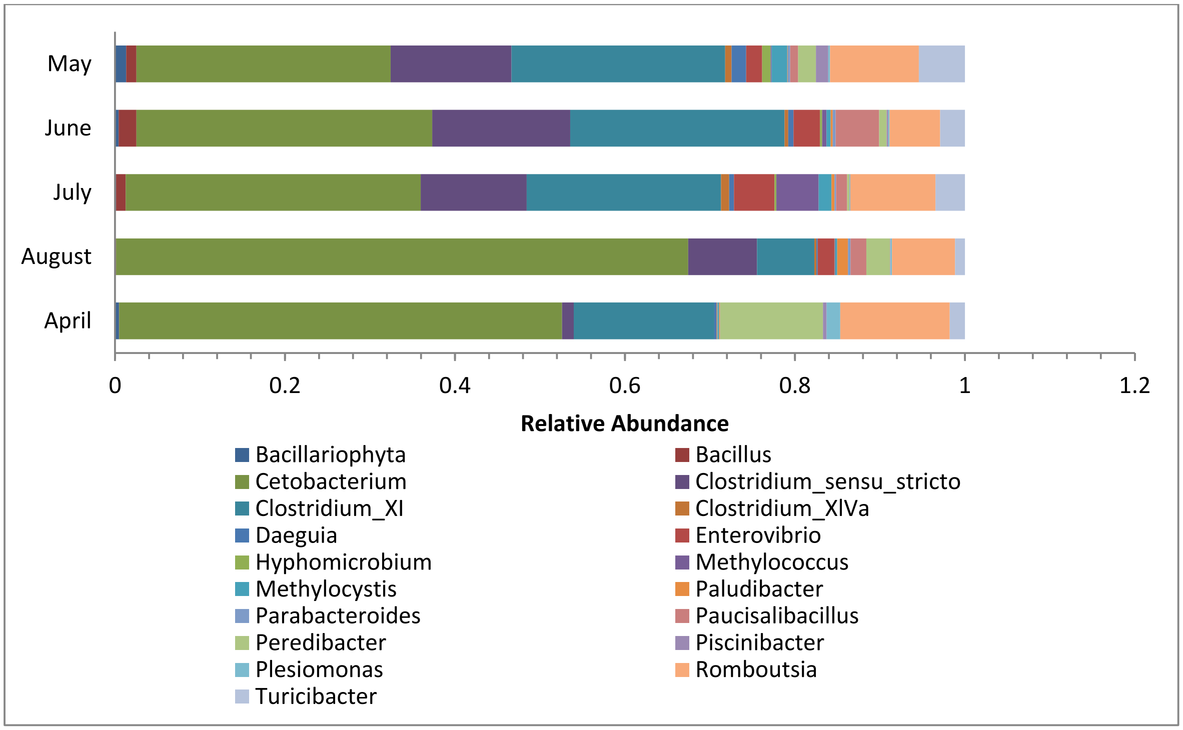

Figure S2. Taxonomic composition of bacterial community of Lake Tana samples at Genus level using stacked plot.

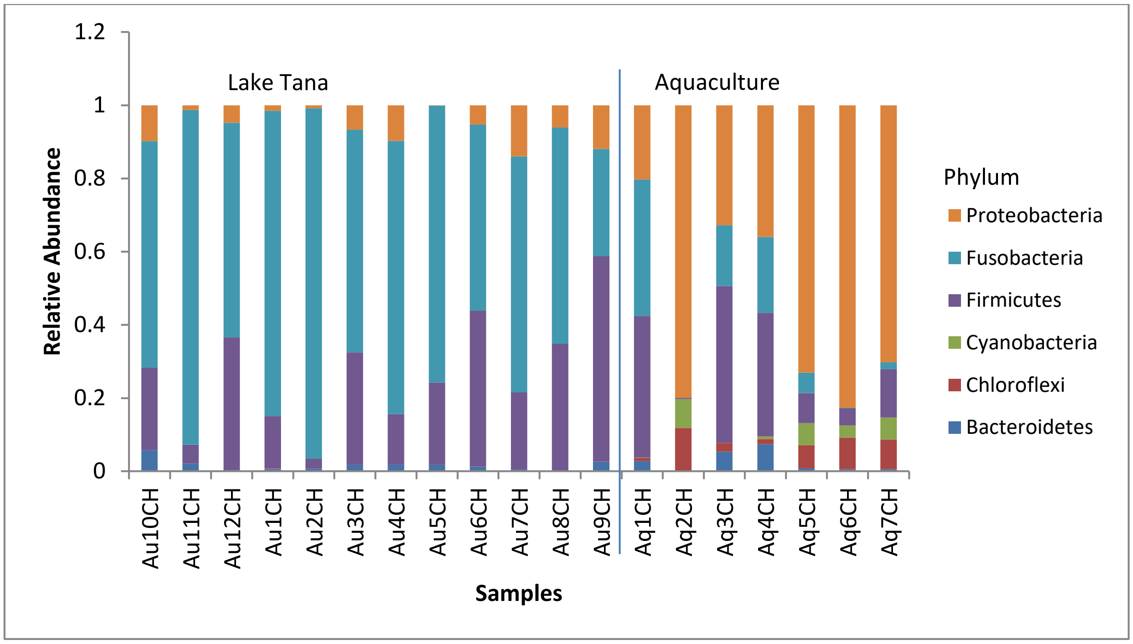

Figure S3. Taxonomic composition of the bacterial community at the phylum level using a stacked plot of aquaculture samples and Lake Tana samples collected in August.

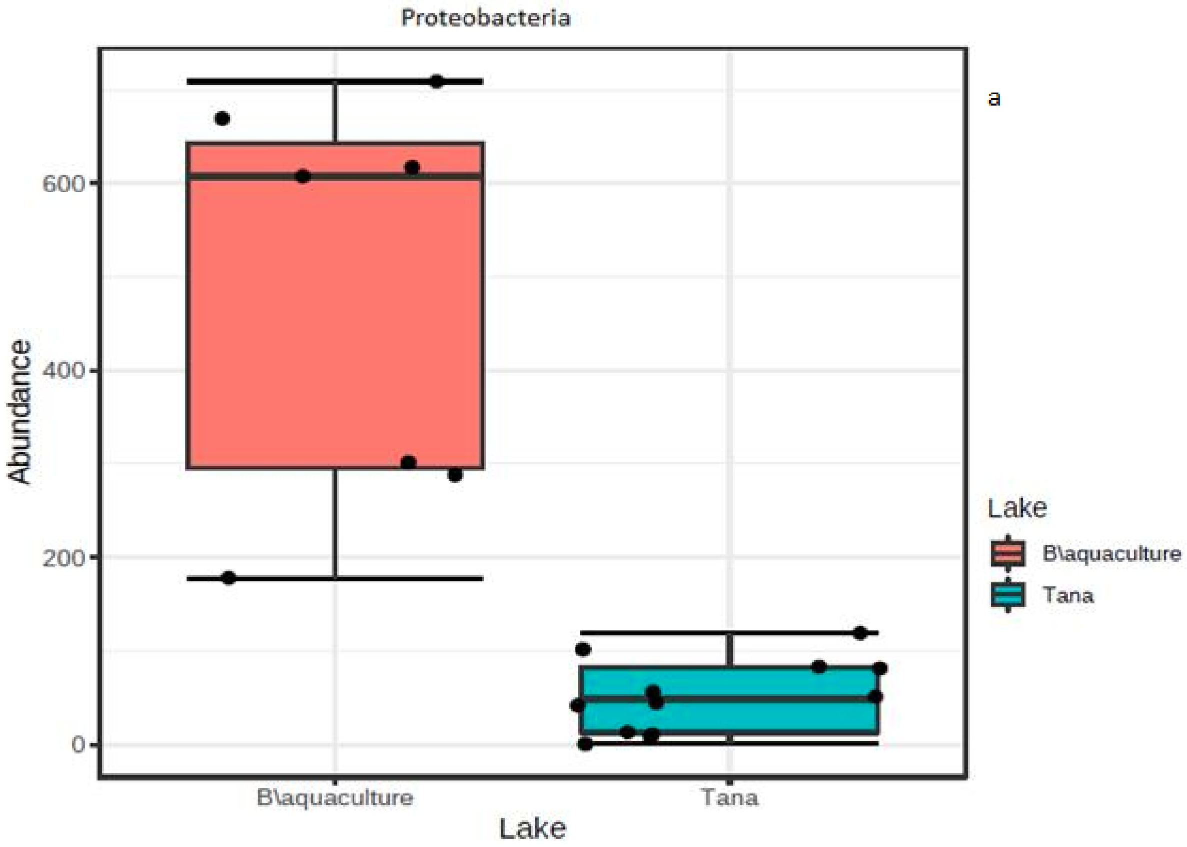

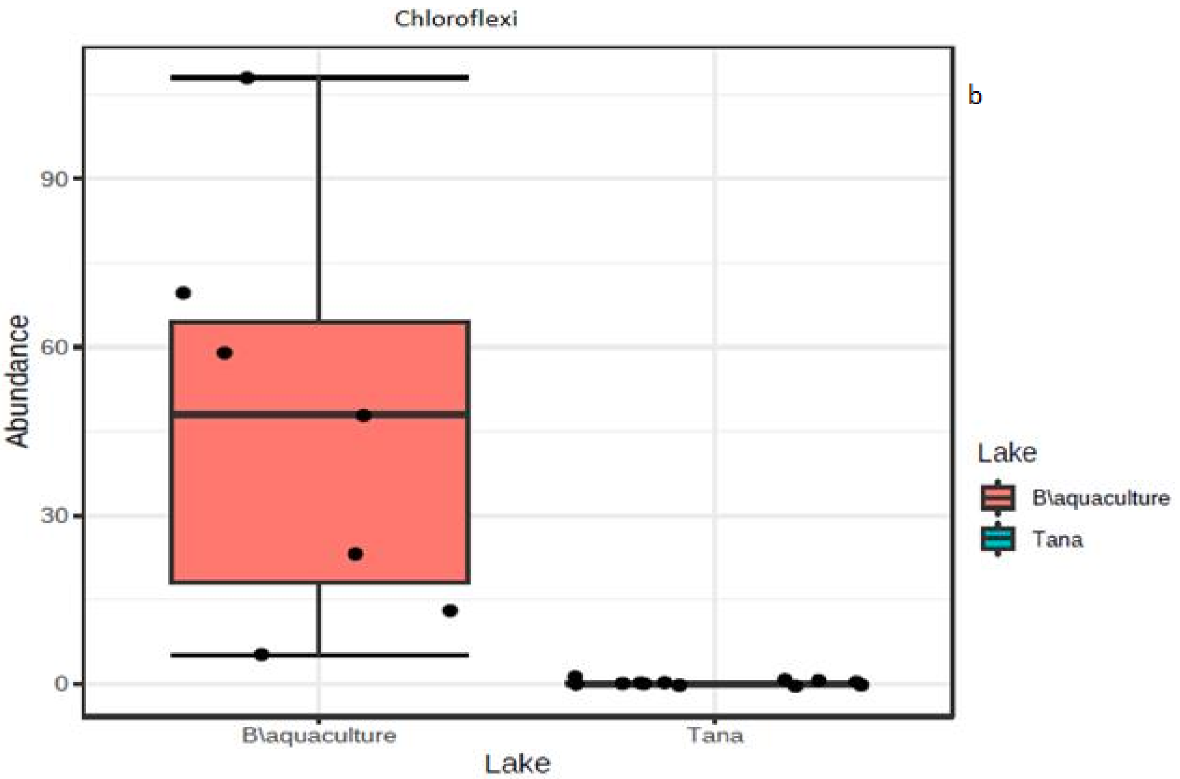
Chloroflexi
b
Abundance
90
60
30
0
B\aquaculture
Tana
Lake
Lake
B\aquaculture
Tana

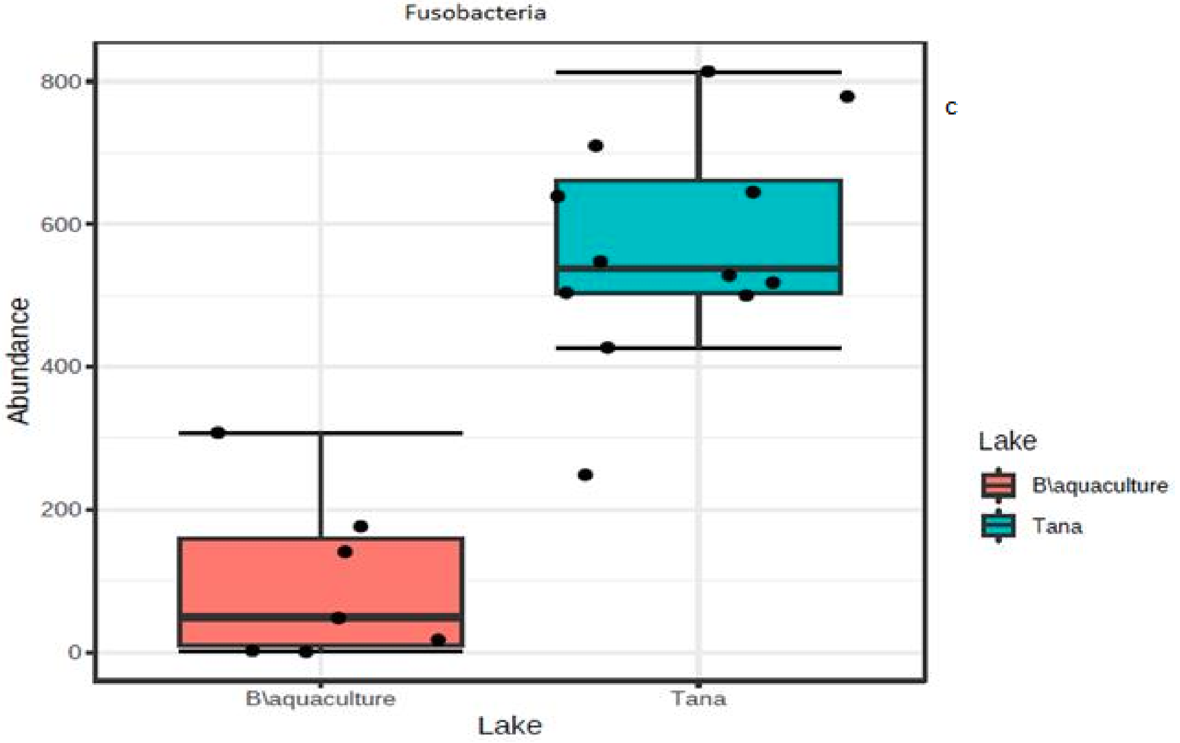
Fusobacteria
c
Abundance
800
600
400
200
0
B\aquaculture
Tana
Lake
Lake
B\aquaculture
Tana

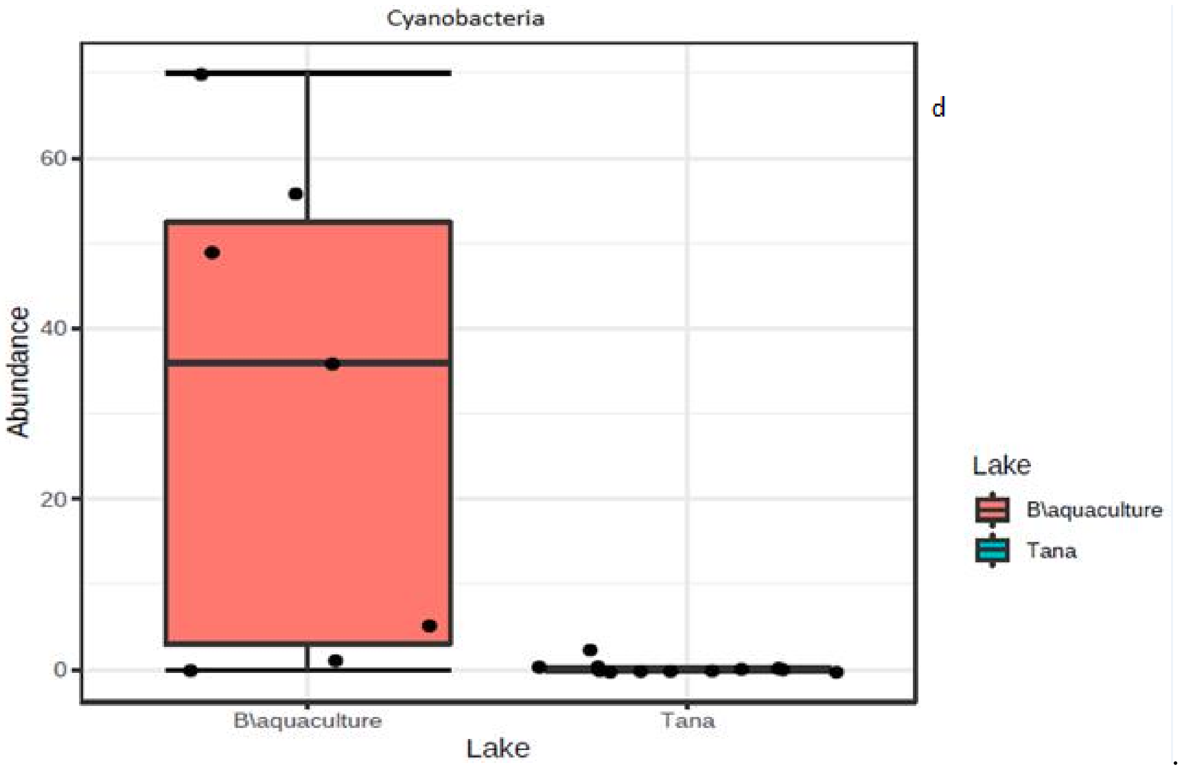

Figure S4. Important features identified by Univariate nonparametric analysis (Mann-Whitney test) at Phylum level from comparison of Bahir Dar aquaculture facility center and Lake Tana samples. Features are considered to be significant based on their adjusted p-value (cutoff = 0.05). (**a**) Phylum Proteobacteria (**b**) Phylum Chloroflexi (**c**) Phylum Fusobacteria and (**d**) Phylum Cyanobacteria

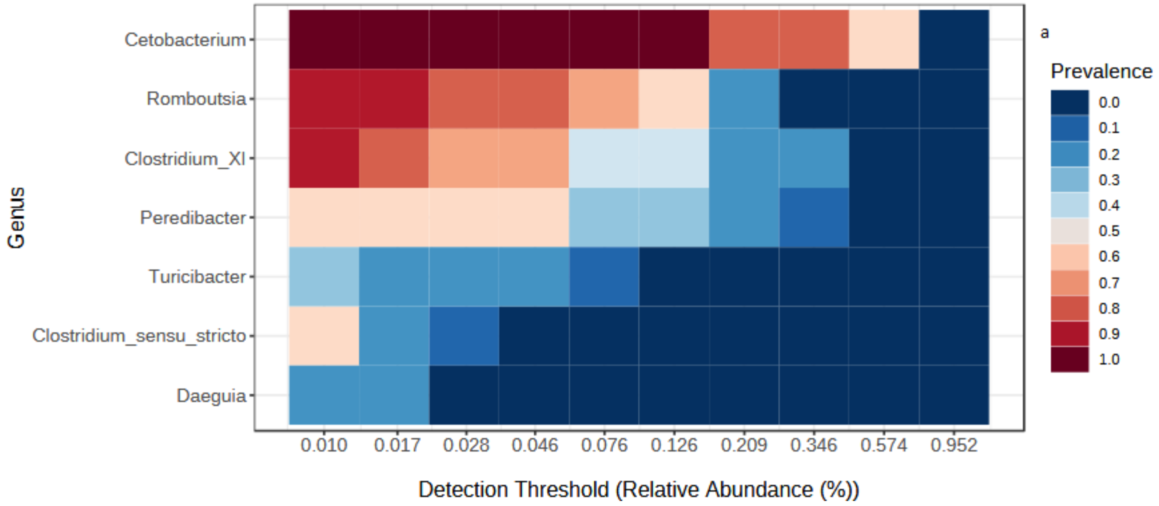

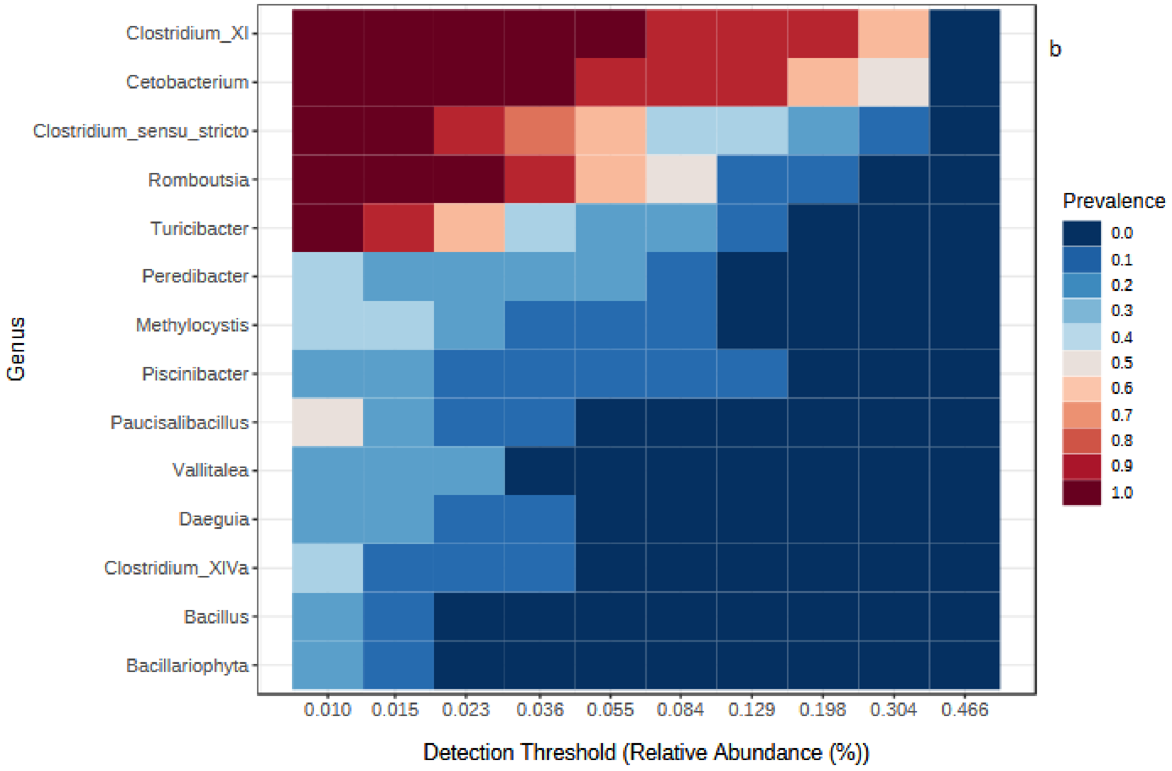
b
Genus
Clostridium_XI
Cetobacterium
Clostridium_sensu_stricto
Romboutsia
Turicibacter
Peredibacter
Methylocystis
Piscinibacter
Paucisalibacillus
Vallitalea
Daeguia
Clostridium_XlVa
Bacillus
Bacillariophyta
0.010
0.015
0.023
0.036
0.055
0.084
0.129
0.198
0.304
0.466
Detection Threshold (Relative Abundance (%))
Prevalence
0.0
0.1
0.2
0.3
0.4
0.5
0.6
0.7
0.8
0.9
1.0

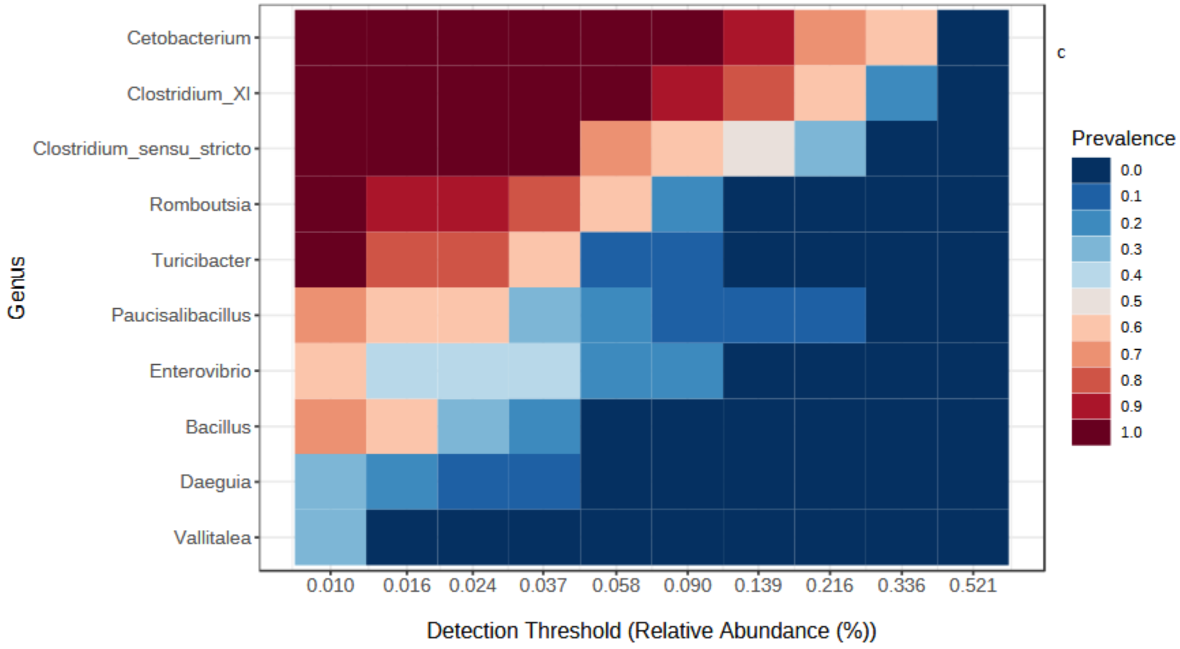
c
Genus
Cetobacterium
Clostridium_XI
Clostridium_sensu_stricto
Romboutsia
Turicibacter
Paucisalibacillus
Enterovibrio
Bacillus
Daeguia
Vallitalea
0.010
0.016
0.024
0.037
0.058
0.090
0.139
0.216
0.336
0.521
Detection Threshold (Relative Abundance (%))
Prevalence
0.0
0.1
0.2
0.3
0.4
0.5
0.6
0.7
0.8
0.9
1.0

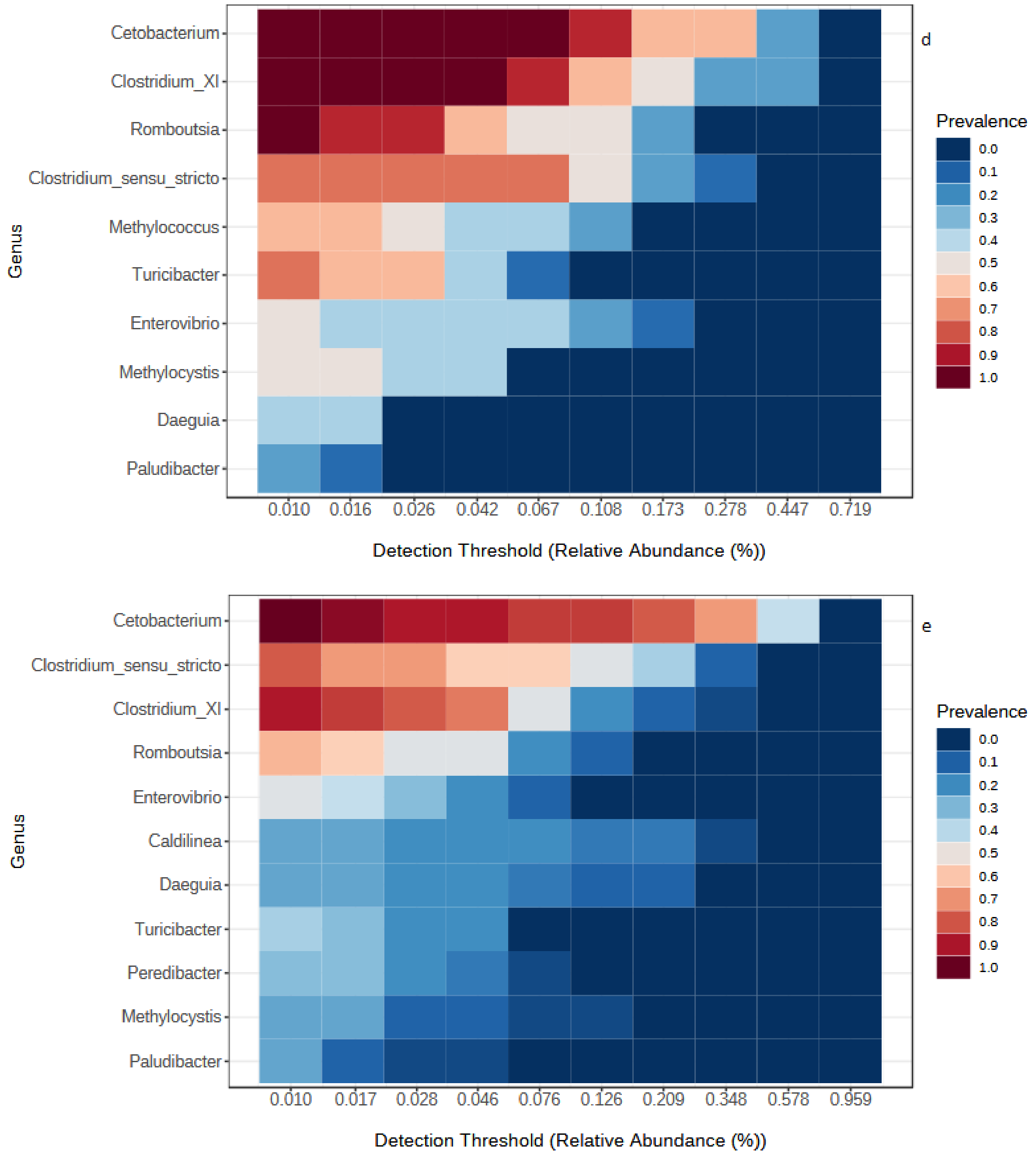

Figure S5. Core microbiota analysis of Lake Tana samples at Genus level for each sampling months. (**a**) April (**b**) May (**c**) June (**d**) July (**e**) August.

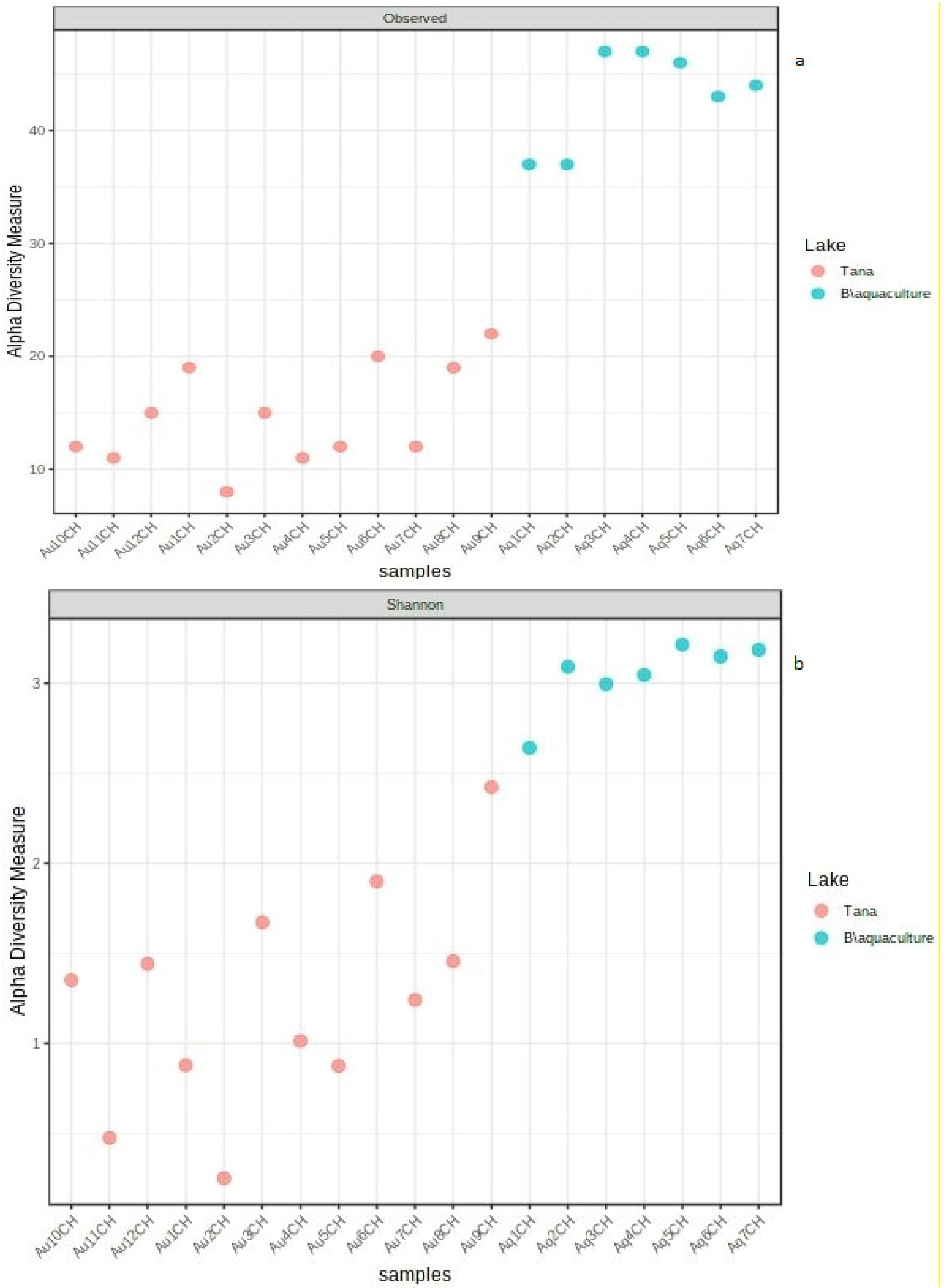
Observed
a
Alpha Diversity Measure
10
20
30
40
samples
Au10CH
Au11CH
Au12CH
Au1CH
Au2CH
Au3CH
Au4CH
Au5CH
Au6CH
Au7CH
Au8CH
Au9CH
Aq1CH
Aq2CH
Aq3CH
Aq4CH
Aq5CH
Aq6CH
Aq7CH
Lake
Tana
Shannon
b
1
2
3

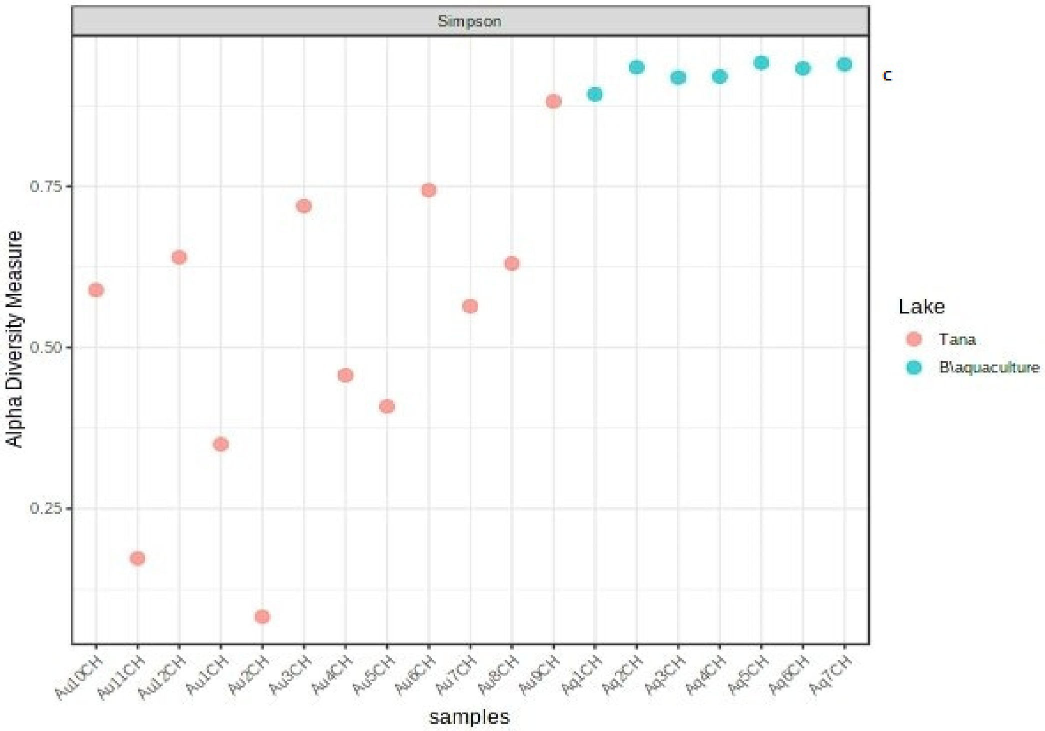

Figure S6. Alpha diversity measured at the OTU level across all the samples for comparison of Lake Tana and the Bahir Dar aquaculture facility centre. Each sample is coloured based on the sources of the samples. (**a**) Observed, (**b**) Shannon index, and (**c**) Simpson index.

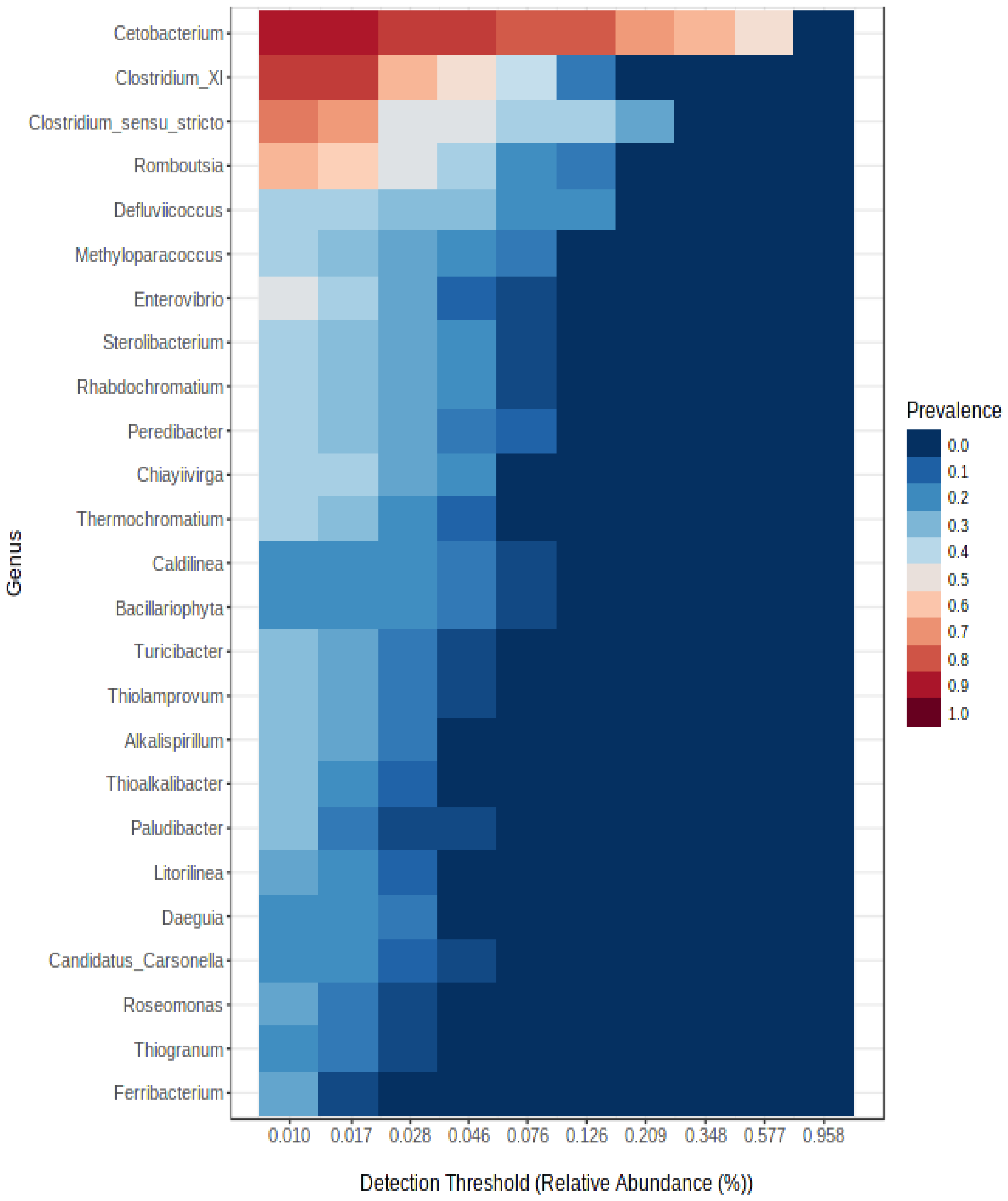

Figure S7. Core microbiota analysis of Lake Tana and aquaculture facility center at Genus level.

Table S1. Important features identified by Univariate nonparametric analysis (Mann-Whitney test) at Genus level from Lake Tana samples.

Genera	***P-Values***
Bacillus	*0.00036*
Paucisalibacillus	*0.001016*
Cetobacterium	*0.001407*
Paludibacter	*0.001586*
Clostridium_XI	*0.002542*
Clostridium_sensu_stricto	*0.0072*
Peredibacter	*0.008173*
Enterovibrio	*0.01257*
Turicibacter	*0.013208*
Clostridium_XlVa	*0.019556*
Bacillariophyta	*0.034771*
Daeguia	*0.041247*

Table S2. Important features identified by Univariate nonparametric analysis (Mann-Whitney test) at Genus level from comparison of Lake Tana and Bahir Dar aquaculture facility center.

Genera	***P-Values***
Desulfatitalea	*4.97E-05*
Ferribacterium	*5.02E-05*
Alkalispirillum	*5.07E-05*
Chiayiivirga	*5.07E-05*
Defluviicoccus	*5.07E-05*
Sterolibacterium	*5.07E-05*
Thioalkalibacter	*5.07E-05*
Thiolamprovum	*5.07E-05*
Litorilinea	*9.37E-05*
Methyloparacoccus	*9.45E-05*
Rhabdochromatium	*9.45E-05*
Thermochromatium	*9.45E-05*
Caldilinea	*0.000148*
Candidatus_Carsonella	*0.000274*
Thermodesulforhabdus	*0.000276*
Paludibacter	*0.000312*
Romboutsia	*0.0004*
Cetobacterium	*0.000616*
Roseomonas	*0.000632*
Thiogranum	*0.000632*
Bacillariophyta	*0.0009*
Peredibacter	*0.002053*
Rhizobium	*0.003377*
Acetobacteroides	*0.003506*
Wandonia	*0.005362*
Daeguia	*0.006198*
Rhodoligotrophos	*0.006601*
Methylocystis	*0.011922*
Parabacteroides	*0.018822*
Methylococcus	*0.030701*
